# DeepInsight-Net: a CBAM-enhanced ResNet50 framework with focal loss for robust cervical cancer classification on multi-center datasets

**DOI:** 10.3389/fmed.2026.1783634

**Published:** 2026-05-21

**Authors:** Elif İlgazi Kılıç, Şafak Kılıç

**Affiliations:** 1 Kayseri City Hospital, Department of Obstetrics and Gynecology, Kayseri, Türkiye; 2 Department of Computer Science, CHART Laboratory, University of Nottingham, Nottingham, United Kingdom; 3 Faculty of Engineering, Architecture and Design, Department of Software Engineering, Kayseri University, Kayseri, Türkiye

**Keywords:** CBAM, cervical cancer, deep learning, focal loss, Pap smear, ResNet50

## Abstract

**Background:**

Cervical cancer remains one of the leading causes of gynecological mortality worldwide, largely due to the limitations of manual cytological screening, which is time-consuming and susceptible to inter-observer variability. Although deep learning has demonstrated strong potential for automating cervical cytopathology, existing Convolutional Neural Network (CNN) models are hindered by two critical challenges: *spatial irrelevance*, where diagnostically meaningful nuclear regions are overshadowed by background artifacts such as blood and mucus, and severe *class imbalance*, where the dominance of normal cells impedes the accurate learning of rare dysplastic patterns.

**Methods:**

To address these limitations, we propose DeepInsight-Net, a robust three-stage deep learning framework for cervical cell classification. The core architecture integrates Convolutional Block Attention Modules (CBAM) into a ResNet50 backbone to enhance spatial and channel-wise feature discrimination, enabling the network to selectively emphasize nuclear and nuclear–cytoplasmic boundary regions while suppressing irrelevant background noise. To further mitigate class imbalance, the conventional cross-entropy loss is replaced with Focal Loss, which dynamically down-weights easily classified samples and prioritizes hard, misclassified instances during training.

**Results:**

Extensive experiments conducted on the benchmark SiPaKMeddataset demonstrate that DeepInsight-Net achieves a state-of-the-art classification accuracy of 99.63%, outperforming 15 competitive deep learning models, including EfficientNet-B6 and DenseNet169. Moreover, cross-dataset generalization experiments on an independent Liquid-Based Cytology (LBC) dataset yield an accuracy of 98.62%, confirming the robustness and domain adaptability of the proposed framework. Visual interpretability analyses using Grad-CAM and t-SNE reveal that the model consistently focuses on biologically relevant cellular regions, supporting the reliability of its predictions.

**Conclusion:**

The proposed DeepInsight-Net effectively addresses spatial irrelevance and class imbalance in cervical cytology analysis through attention-guided feature learning and loss re-weighting. The strong performance across multiple datasets, combined with transparent visual explainability, highlights its potential as a reliable computer-aided diagnosis (CAD) tool for supporting cervical cancer screening in real-world clinical settings.

## 1 Introduction

Cervical cancer represents a profound global health crisis, ranking as the fourth most frequently diagnosed malignancy and the fourth leading cause of cancer death in women worldwide. According to the latest GLOBOCAN statistics, approximately 604,000 new cases and 342,000 deaths were reported in a single year, with nearly 90% of these fatalities occurring in low- and middle-income countries (LMICs) (1, 2). The primary strategy for mitigating this burden is early screening via the Papanicolaou (Pap) smear test, which aims to detect pre-cancerous lesions (cervical intraepithelial neoplasia) before they progress to invasive carcinoma. While the widespread adoption of Pap screening has significantly reduced mortality rates in developed nations, manual interpretation remains fraught with challenges. The analysis is labor-intensive, monotonous, and highly subjective, leading to inter-observer variability and a false-negative rate ranging from 20% to 50% due to human fatigue and the complexity of distinguishing abnormal cells within overlapped clusters (3, 4).

In recent years, the digitization of pathology slides has paved the way for Computer-Aided Diagnosis (CAD) systems, where Deep Learning (DL) has emerged as a transformative force. Within this domain, attention mechanisms have become indispensable, proving their efficacy across a spectrum of tasks—from precise segmentation in hybrid networks like FocusGate-Net (5) to complex classification problems. Despite these advancements, however, standard Convolutional Neural Networks (CNNs) still face significant limitations when applied to raw cytopathological data. Deep Learning (DL), particularly CNNs, has emerged as the state-of-the-art methodology for automated cervical cytopathology (6). Models such as VGG, Inception, and ResNet have achieved remarkable success in classifying single cells. However, despite these advancements, standard CNN architectures face two persistent limitations when applied to clinical cytology:

*1. The Challenge of Spatial Irrelevance and Noise:* In Pap smear slides, diagnostic features are often localized in minute regions, specifically the nucleus (chromatin texture) and the nuclear-cytoplasmic boundary (N/C ratio). Standard CNNs utilize global pooling operations that treat background artifacts such as mucus, blood, inflammatory cells, and overlapping cytoplasm with the same importance as the nucleus (7). This lack of “attention” often leads the model to learn irrelevant background features, reducing generalization capability on unseen data.

*2. The Class Imbalance Problem:* Medical datasets are inherently imbalanced; the number of normal (Superficial/Intermediate) cells vastly outnumbers abnormal (Dysplastic) cells. Training a model with the standard Cross-Entropy (CE) loss function on such skewed data results in a bias toward the majority class. The accumulated gradients from thousands of easy “normal” examples overwhelm the rare but critical “abnormal” signals, causing the model to achieve high overall accuracy but suboptimal sensitivity for high-grade lesions (8).

To bridge these significant research gaps, we propose DeepInsight-Net, a robust and clinically applicable framework. Unlike heavy Vision Transformer (ViT) models that require massive datasets, or standard CNNs that lack spatial awareness, our approach introduces a synergistic integration of attention mechanisms and advanced loss optimization. The specific contributions of this study are as follows:

1. *Architectural Novelty (CBAM-ResNet50):* We integrate the Convolutional Block Attention Module (CBAM) (9) into the residual blocks of the ResNet50 backbone. This dual-attention mechanism mimics the human pathologist's workflow: the *Channel Attention* module selects “what” features are biologically relevant (e.g., chromatin density), while the *Spatial Attention* module learns “where” to focus (e.g., the nucleus), effectively suppressing background noise.
2. *Mathematical Novelty (Focal Loss Integration):* We address the severe class imbalance not by synthetic oversampling, but by reshaping the objective function. We implement Focal Loss (8), which dynamically down-weights the contribution of easy samples and focuses the model's training on “hard” negatives (misclassified dysplastic cells). This ensures high sensitivity for rare classes without sacrificing specificity.
3. *Comprehensive Validation & Robustness:* Beyond standard metrics, we validate our model's robustness using Test-Time Augmentation (TTA) and demonstrate its interpretability through Grad-CAM visualizations. We provide a rigorous evaluation on the challenging SiPaKMed dataset and generalization analysis on the liquid-based cytology (LBC) domain.

## 2 Related work

The landscape of automated cervical cancer screening has evolved significantly, transitioning from traditional machine learning techniques relying on handcrafted features to sophisticated end-to-end deep learning architectures. This section reviews the progression of these methodologies, highlighting recent advancements and identifying existing research gaps.

### 2.1 Traditional machine learning approaches

Before the dominance of deep learning, CAD systems were predicated on the explicit extraction of “handcrafted” features. These methodologies required domain experts to define mathematical descriptors for cell morphology, texture, and chromatin distribution.

Early research focused on segmenting the nucleus and cytoplasm to calculate ratios such as the Nucleus-to-Cytoplasm (N:C) ratio, a critical biomarker for malignancy. Plissiti et al. were pivotal in this era, introducing the SIPaKMeD dataset a benchmark still in heavy use in 2025 and establishing baselines using spectral clustering combined with Support Vector Machines (SVM) (10). Their approach relied on the assumption that dysplastic cells exhibit distinct high-frequency texture components and enlarged nuclei compared to healthy squamous epithelium. While these methods provided foundational insights, they were fundamentally limited by the quality of the initial segmentation. If the segmentation algorithm failed to separate overlapping cells a common occurrence in conventional Pap smears the downstream classifier would inevitably fail.

While methods utilizing SVMs, k-Nearest Neighbors (k-NN), and Random Forests provided interpretability, they lacked generalization capabilities. Handcrafted features are highly sensitive to staining variations (e.g., Papanicolaou vs. Hematoxylin & Eosin) and acquisition artifacts. Research indicates that while these models could achieve reasonable specificity in controlled datasets, their sensitivity dropped significantly when applied to multi-center data due to the “domain shift” caused by varying laboratory protocols. The rigidity of feature engineering necessitated a shift toward end-to-end learning, where the model learns to identify optimal features directly from pixel data.

### 2.2 The Convolutional Neural Network (CNN) era

The advent of CNNs marked a transition from feature engineering to feature learning. By 2024, CNNs had become the gold standard for cervical cytology classification, with architectures such as ResNet, DenseNet, and EfficientNet serving as the backbone for the majority of diagnostic systems.

Transfer learning remains a dominant strategy in 2024–2025, particularly given the scarcity of large-scale annotated medical datasets (6). Furthermore, recent advancements in training protocols, such as chaotic learning rate scheduling, have been shown to further stabilize the convergence of CNN-based models in medical ultrasound classification (11).

The Residual Network (ResNet) family, particularly ResNet50 and ResNet101, is frequently cited as a robust feature extractor. A 2025 study evaluating 16 pre-trained models on the Herlev and SIPaKMeD datasets identified ResNet50 as a top performer, achieving high validation accuracy through its ability to mitigate the vanishing gradient problem via skip connections (12, 13). Kondo and Ueno (2023) demonstrated that applying TTA with a single ResNet50 backbone could stabilize predictions (12). However, their approach utilized standard Cross-Entropy loss, which treats all samples equally, failing to address the severe class imbalance where rare dysplastic cells are often overshadowed by the majority class.

Densely Connected Convolutional Networks (DenseNet) have shown superiority in scenarios where feature preservation is critical. By connecting each layer to every other layer in a feed-forward fashion, DenseNet maximizes information flow. Recent work in 2025 demonstrated that fine-tuning DenseNet-121 on the SIPaKMeD dataset could achieve up to 100% testing accuracy in five-class distribution tasks, outperforming shallower networks (14).

To further boost performance, researchers have moved toward ensemble learning. Hussain et al. (2020) proposed an aggregation of outputs from multiple CNN backbones (AlexNet, VGG, ResNet) to improve classification on liquid-based cytology images (15). More recent 2025 frameworks have optimized this by integrating “Expert” sub-models that specialize in distinguishing difficult subclasses, such as differentiating between High-grade Squamous Intraepithelial Lesions (HSIL) and Squamous Cell Carcinoma (SCC). For instance, the DeepCervix framework employs hybrid deep feature fusion, combining representations from multiple backbones to achieve accuracies exceeding 99% on the SIPaKMeD dataset (16).

*Computational Cost of Ensembles:* Despite their accuracy, ensemble methods incur prohibitive computational costs. A study utilizing a weighted ensemble of DenseNet201, Xception, and InceptionResNetV2 noted that while the ensemble achieved an accuracy of 96.58% on the Mendeley LBC dataset, the inference time and parameter count rendered it unsuitable for real-time deployment in mobile health (mHealth) settings (17). This limitation has driven two distinct research directions in 2025: the development of lightweight models for edge computing and the integration of attention mechanisms to improve single-model efficiency.

### 2.3 Vision Transformers (ViTs) and hybrid architectures

The most significant architectural development in 2024–2025 is the integration of ViTs into the cervical cancer screening pipeline. While CNNs excel at capturing local spatial hierarchies (edges, textures), they struggle with long-range dependencies contextual relationships between distant parts of an image which are crucial for analyzing cell clusters and tissue architecture.

Pure Transformers often require massive datasets to converge. Consequently, the state-of-the-art (SOTA) has coalesced around hybrid architectures that use CNNs for local feature extraction and Transformers for global context modeling.

*Emara et al*. conducted a comprehensive evaluation of fusion strategies, combining CNNs with ViTs to capture both local and global dependencies (18). Their hybrid models achieved high accuracy but significantly increased model complexity and training time. Similarly, Hong et al. (19) introduced a framework combining Low-Rank Adaptation (LoRA) with ViTs. This approach allows the model to adapt to specific cervical cytology domains with minimal trainable parameters, outperforming traditional ResNets in limited-data scenarios (19).

*Swin Transformers:* The Swin Transformer, with its hierarchical shifted window mechanism, has gained traction for its efficiency. In 2024, hybrid models combining Swin Transformers with DenseNet and VGG backbones were proposed to improve classification on the SIPaKMeD dataset (20). These models address the fixed receptive field limitation of CNNs, allowing the network to “attend” to the relationship between a dysplastic nucleus and the surrounding cytoplasm more effectively than convolution alone.

While hybrids like MaxCerVixT (CNN-based ViT) achieve exceptional accuracies (up to 99.02% on SIPaKMeD) (21), they introduce significant latency. The attention mechanism's quadratic complexity with respect to token length remains a bottleneck. This has led to critical comparisons where simpler, attention-enhanced CNNs (like the proposed DeepInsight-Net) are preferred for practical applications over heavy Transformer stacks.

Addressing the computational bottleneck, a parallel stream of research in 2024–2025 focuses on lightweight models and optimization algorithms.

Architectures like MobileNetV3 and ShuffleNet utilize depth-wise separable convolutions to reduce computation. CACCD-GOADL (2024) combined MobileNetV3 with the Gazelle Optimization Algorithm, achieving 99.38% accuracy with a fraction of the parameters of a ResNet-152 (22). However, Yadav et al. (2024) noted that such metaheuristic-driven frameworks often involve complex multi-stage pipelines that lack the simplicity of end-to-end gradient-based learning (23).

A novel 2025 approach, RL-Cervix.Net, integrates Reinforcement Learning (RL) with lightweight CNNs (EfficientNetV2). The RL agent dynamically adjusts the focus of the model during the screening process, effectively “learning where to look” in a manner analogous to a human screener (24). While promising, the training complexity of RL policies makes convergence difficult compared to supervised attention modules.

Consequently, the state-of-the-art (SOTA) has coalesced around hybrid architectures that use CNNs for local feature extraction and Transformers for global context modeling. Recent comparative evaluations in other medical domains, such as breast and brain tumor classification, have further underscored the trade-offs between pure Transformer architectures and traditional convolutional networks regarding both accuracy and computational efficiency (25, 26).

### 2.4 Attention mechanisms and loss functions

To capture the benefits of global context and structural relationships in medical imaging, researchers have explored various sophisticated architectures. For instance, Kılıç demonstrated the efficacy of a dual-path attention framework combining ConvNeXt and Swin Transformers for blood cell classification (27), while Kalas (2025) utilized graph-based deep learning (GNN) for anatomically informed spinal disease analysis (28).

However, to avoid the high computational overhead of full Transformers in cytopathology, researchers have increasingly adopted modular attention mechanisms and specialized loss functions to recalibrate feature maps efficiently.

- *SE Blocks:* Focus on inter-channel dependencies. A 2025 study on RegNetX demonstrated that adding SE blocks significantly improved F1-scores by focusing the network on relevant feature maps (29).
- *CBAM:* Dogan (2025) utilized CBAM within a ResNet-based autoencoder (AutoEffFusionNet), showing that spatial attention is critical for localizing small, dysplastic nuclei within large cytoplasm regions (30). The spatial attention component helps the model effectively “ignore” the background, addressing the issue of *spatial irrelevance*.

Medical datasets are inherently imbalanced. Standard Cross-Entropy (CE) loss is ill-suited for this, as the massive number of “easy” negative examples (normal cells) dominates the gradient. Lalitha et al. and other recent benchmarks indicate that models trained with Focal Loss achieve higher sensitivity for minority classes compared to those trained with CE or weighted CE (31, 32). Focal Loss adds a modulating factor to the CE loss to down-weight easy examples and focus training on hard negatives.

### 2.5 Research gap and motivation

Despite these advancements, a critical gap remains in the literature of 2024–2025. The field is largely bifurcated into two extremes:

1. *High-Complexity Hybrids:* Models like TransNet and ensemble ViTs achieve SOTA accuracy but are computationally heavy and difficult to interpret.
2. *Lightweight Models with Trade-offs:* Models like MobileNetV3 are efficient but often struggle to simultaneously handle *spatial irrelevance* (background noise) and *class imbalance* without resorting to complex auxiliary optimization (like metaheuristics or RL).

Existing lightweight models using standard loss functions often fail to detect rare dysplastic cells in noisy environments. Our proposed DeepInsight-Net addresses this by integrating the lightweight CBAM attention module directly into the ResNet backbone and employing Focal Loss. This combination offers a robust, end-to-end solution that:

- Uses Spatial Attention to filter out mucus and blood (Spatial Irrelevance).
- Uses Focal Loss to prevent the majority class from overwhelming the gradient (Class Imbalance).
- Maintains the efficiency of a single backbone, avoiding the latency of ensembles or Transformers.

Table 1 summarizes these comparisons against the most recent state-of-the-art.

**Table 1:** Enhanced comparison of DeepInsight-Net with state-of-the-art literature (2018–2025) including performance metrics.

Study (year)	Methodology	Dataset	Result (%)	Limitation addressed by ours
Plissiti et al. (10)	Spectral Clustering + SVM	SiPaKMed	95.00	Relies on handcrafted features; we use end-to-end DL.
Hussain et al. (15)	Ensemble of Multiple CNNs	LBC	98.23	High computational cost; we use a single efficient backbone.
Kondo et al. (12)	ResNet50 + TTA	SiPaKMed	98.15	Uses standard Cross-Entropy Loss; we use Focal Loss for imbalance.
Emara et al. (18)	CNN + ViT Fusion	Multi-Dataset	99.10	Complex hybrid architecture; our CBAM is lightweight and effective.
Yadav et al. (23)	DL + Metaheuristics	SiPaKMed	99.38	Multi-stage optimization; we use direct gradient-based learning.
Nour et al. (22)	MobileNetV3 + Gazelle Opt.	Herlev	99.38	Relies on complex bio-inspired optimization.
Hong et al. (19)	ViT + LoRA	Multi-Dataset	98.50	Transformer-based complexity; we avoid self-attention overhead.
Muksimova et al. (24)	RL + EfficientNetV2	Multi-Dataset	98.92	High training complexity due to RL policy convergence.
Dogan (30)	ResNet + CBAM (Autoencoder)	SiPaKMed	97.40	Unsupervised focus; we apply CBAM in a supervised context.
Proposed Method	CBAM-ResNet50 + Focal Loss	SiPaKMed	99.63	Simultaneously addresses spatial noise via Attention and class imbalance via Focal Loss.

## 3 Methodology

**Figure 1:**
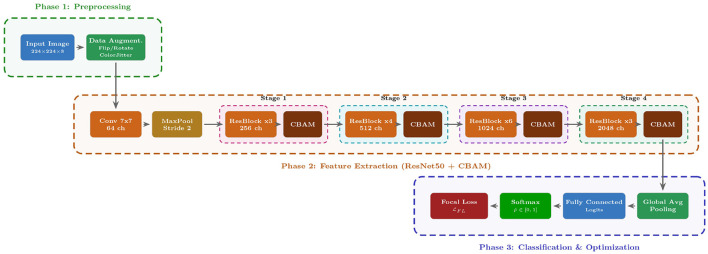
Schematic overview of the DeepInsight-Net framework. The architecture integrates CBAM attention blocks after each ResNet stage to refine feature representations. The final classification is optimized using Focal Loss to address class imbalance.

In this study, we propose DeepInsight-Net, a specialized deep learning framework engineered to tackle the tri-fold challenges of cervical cytopathology: (1) high intra-class variance and inter-class similarity, (2) the scarcity of annotated medical data, and (3) severe class imbalance. As depicted in the schematic overview in Figure 1, our methodology is structured into a cohesive pipeline starting from data acquisition to optimization.

### 3.1 Materials and datasets

The foundation of any robust deep learning framework lies in the quality and diversity of its training data. To ensure clinical relevance and evaluate cross-domain generalization, we employed two distinct cytopathological datasets: SiPaKMed (Conventional Smear) and LBC (Liquid Based Cytology). The datasets were explicitly divided into training, validation, and test subsets to ensure a reliable evaluation protocol. For each dataset, approximately 70% of the data was allocated for training, 10% for validation, and the remaining 20% for testing. The validation set was used to monitor model performance and prevent overfitting during training, while the test set was kept completely unseen for the final performance evaluation.

#### 3.1.1 Primary benchmark: SiPaKMed dataset

The SiPaKMed dataset (10) was utilized for training and primary validation. It contains 4,049 manually cropped single-cell images acquired via CCD cameras adapted to optical microscopes. The dataset is categorized into five classes based on cellular morphology:

- *Superficial-Intermediate:* Normal squamous cells characterized by small pyknotic nuclei and abundant, transparent cytoplasm.
- *Parabasal:* Immature cells found in atrophic smears, often confused with metaplastic cells due to their dense cytoplasm and relatively larger nuclei.
- *Koilocytotic:* Cells exhibiting perinuclear halos, a distinct cytopathic effect of Human Papillomavirus (HPV) infection (Low-grade lesion).
- *Dyskeratotic:* Cells with abnormal keratinization, pleomorphic shapes, and hyperchromatic nuclei (High-grade lesion).
- *Metaplastic:* Benign cells with dense cytoplasm that can mimic dysplastic abnormalities, serving as a critical distractor class.

This dataset presents significant challenges due to overlapping cells, inflammatory background noise, and blood artifacts typical of conventional smears (Figure 1a).

#### 3.1.2 Generalization benchmark: LBC dataset

**Figure 2:**
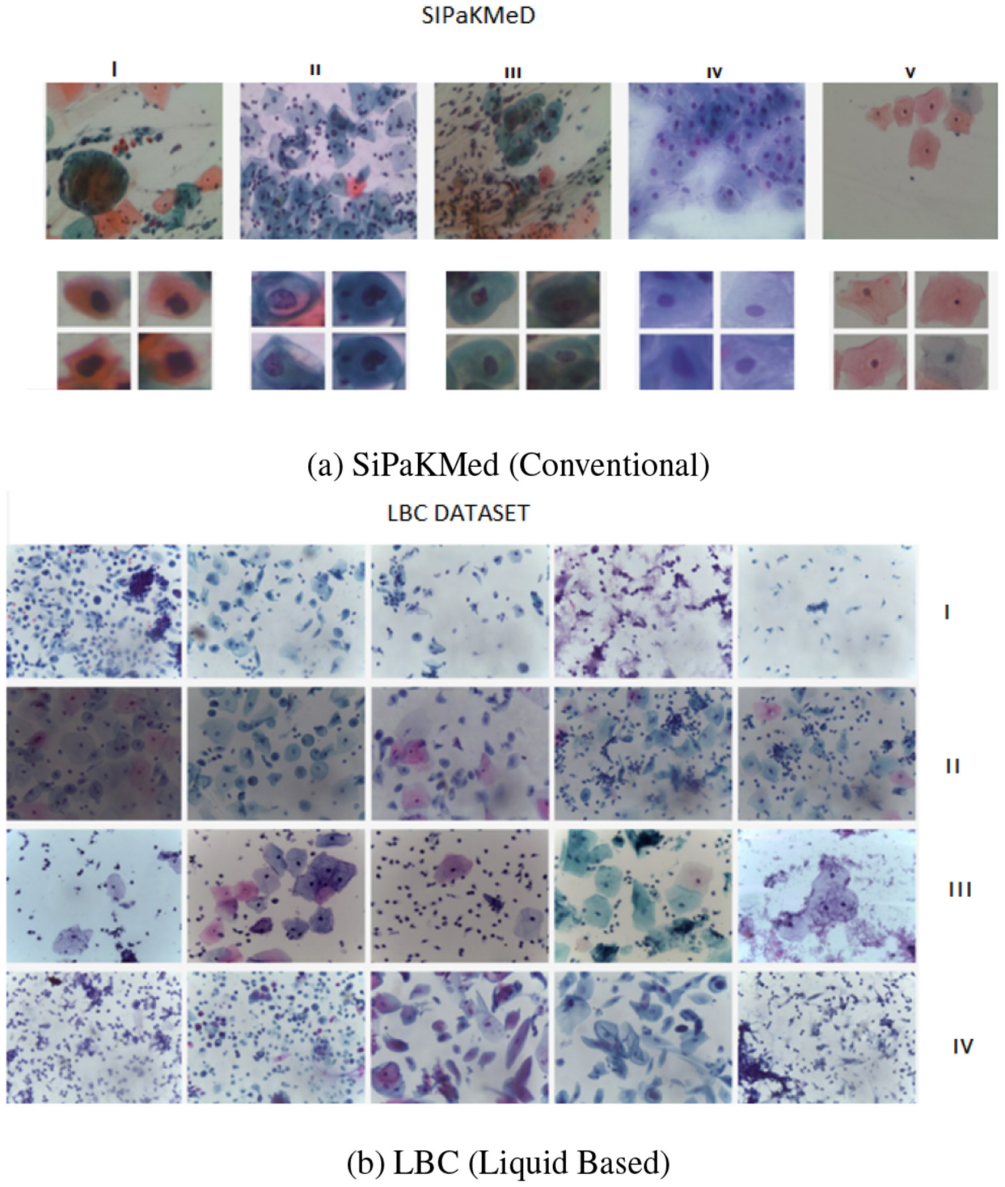
Representative samples from the datasets. **(a)** SiPaKMed dataset images with complex backgrounds, categorized into five morphological classes: I: Dyskeratotic, II: Koilocytotic, III: Metaplastic, IV: Parabasal, and V: Superficial-Intermediate. **(b)** LBC dataset images with clearer backgrounds and monolayer dispersion, showing four clinical categories: I: NILM, II: LSIL, III: HSIL, and IV: SCC.

To assess the robustness of our model against domain shifts, we utilized the Mendeley LBC dataset (15). Unlike conventional smears, LBC utilizes a filtration process that removes obscuring material like blood and mucus, resulting in a cleaner, monolayer background (Figure 2b). This dataset follows the Bethesda System with four classes: Negative for Intraepithelial Malignancy (NIM), Low-grade (LSIL), High-grade (HSIL), and Squamous Cell Carcinoma (SCC).

### 3.2 Overview of the proposed framework

Given the raw nature of the microscopic images and the inherent class imbalance, a direct application of standard CNNs would be suboptimal. To address these specific challenges effectively, we designed DeepInsight-Net as a cohesive three-stage pipeline (see Figure 1):

- *Stage 1:* Standardizes the heterogeneous input data via adaptive preprocessing.
- *Stage 2:* Extracts deep discriminative features using the CBAM-enhanced backbone.
- *Stage 3:* Optimizes the learning process to focus on hard-to-classify examples via Focal Loss.

### 3.3 Stage 1: adaptive pre-processing and augmentation

**Figure 3:**
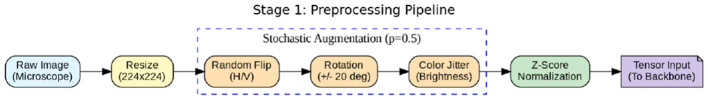
Stage 1: the adaptive pre-processing pipeline. Raw microscopic images undergo resizing, stochastic augmentation, and Z-score normalization before entering the backbone network.

The quality of input data directly dictates the upper bound of model performance. Variations in staining intensity (e.g., Hematoxylin & Eosin) and illumination are inevitable in multi-center datasets. To standardize the input domain, we implemented a robust preprocessing pipeline (Figure 3).

#### 3.3.1 Bicubic resizing and normalization

Input images *I*_*raw*_ were first resized to a fixed resolution of 224 × 224 pixels using bicubic interpolation. We selected bicubic over bilinear interpolation as it preserves high-frequency edge details—crucial for defining the nuclear boundary—by utilizing a 4 × 4 pixel neighborhood. Subsequently, Z-score normalization was applied to facilitate stable gradient convergence. Let *I*(*x, y, c*) denote the pixel value at position (*x, y*) for channel *c*. The normalized pixel value $I_{norm}^{\prime }$ is computed as shown in Equation 1:


$$\begin{array}{l} I_{norm}^{\prime }\left(x,y,c\right)=\frac{I\left(x,y,c\right)-\mu_{c}}{\sigma_{c}} \end{array}$$ (1)

where μ_*c*_ and σ_*c*_ represent the channel-wise mean and standard deviation of the ImageNet dataset. This transfer of statistical properties ensures that the pre-trained backbone receives data in a familiar distribution (33).

#### 3.3.2 Stochastic online augmentation

To combat the “small data” problem and prevent overfitting, we employed a stochastic augmentation strategy. During training, each image undergoes a transformation *T*(·) with probability *p* = 0.5. The augmentation space includes:

- *Geometric Invariance:* Random Horizontal/Vertical flips and Rotations (θ∈[−20°, 20°]) model the arbitrary orientation of cells on the slide.
- *Photometric Robustness:* Color Jittering involves random perturbations in brightness (*B*) and contrast (*C*) within a range of ±10%. This simulates varying microscope light intensities and staining variations (34).

### 3.4 Stage 2: the DeepInsight architecture (CBAM-ResNet50)

The core of our framework is the CBAM-Enhanced ResNet50 backbone. Similar attention-driven bottleneck architectures have recently demonstrated robust performance in complex medical segmentation tasks, validating the efficacy of integrating modular attention blocks into deep backbones to suppress irrelevant noise (35). While ResNet50 provides a strong foundation via residual learning, standard convolutional operations are spatially agnostic; they treat background noise (mucus, blood) with the same importance as the nucleus. In cytopathology, however, diagnostic information is highly localized.

**Figure 4:**
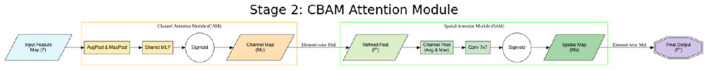
Stage 2: detailed structure of the CBAM Integration. The module sequentially applies Channel Attention (to select meaningful features) and Spatial Attention (to focus on the nucleus).

To address this, we integrated the Convolutional Block Attention Module (CBAM) (9) into the bottleneck blocks. As detailed in Figure 4, CBAM sequentially infers attention maps along two dimensions: Channel and Spatial. This sequential arrangement has been shown to outperform parallel arrangements in preserving feature hierarchy.

#### 3.4.1 Channel Attention Module (CAM): “what to look for”

This module focuses on selecting meaningful feature maps (e.g., specific texture detectors for chromatin). Unlike Squeeze-and-Excitation (SE) networks which use only Global Average Pooling (GAP), CBAM utilizes both GAP and Global Max Pooling (GMP). The rationale is that Average Pooling captures general statistics, whereas Max Pooling captures distinctive object features (e.g., high-intensity pixel clusters in hyperchromatic nuclei). The channel attention map $M_{c}\in {\mathbb{R}}^{C\times 1\times 1}$ is computed as shown in Equation 2:


$$\begin{array}{l} M_{c}\left(F\right)=\sigma \left(MLP\left(AvgPool\left(F\right)\right)+MLP\left(MaxPool\left(F\right)\right)\right) \end{array}$$ (2)

where σ denotes the sigmoid activation function, and *MLP* is a shared Multi-Layer Perceptron. The refined feature *F*′ is obtained by element-wise multiplication shown in Equation 3:


$$\begin{array}{l} F^{\prime }=M_{c}\left(F\right)⊗F \end{array}$$ (3)

#### 3.4.2 Spatial Attention Module (SAM): “where to look”

After channel refinement, the Spatial Attention Module (SAM) focuses on localizing the informative regions (ROIs). It aggregates channel information by applying average-pooling and max-pooling operations along the channel axis, generating two 2D maps. These are then convolved with a large receptive field kernel (7 × 7) to generate the spatial map *M*_*s*_, shown in Equation 4:


$$\begin{array}{l} M_{s}\left(F^{\prime }\right)=\sigma \left(f^{7\times 7}\left(\left[AvgPool\left(F^{\prime }\right);MaxPool\left(F^{\prime }\right)\right]\right)\right) \end{array}$$ (4)

The final refined feature map *F*″ is obtained shown in Equation 5:


$$\begin{array}{l} F^{\prime \prime }=M_{s}\left(F^{\prime }\right)⊗F^{\prime } \end{array}$$ (5)

This mechanism acts as a “soft” ROI selector, suppressing background noise while amplifying the nuclear signal, which is critical for accurate grading of dysplasia.

### 3.5 Stage 3: optimization via hard example mining

The final stage addresses the chronic class imbalance problem in medical datasets. In SiPaKMed, normal cells vastly outnumber dysplastic cells. Training with standard Cross-Entropy (CE) loss causes the model to be overwhelmed by easy-negative examples (background or clear normal cells), leading to high accuracy but low sensitivity for rare, critical classes. The standard CE loss is defined as *CE*(*p*_*t*_) = −log(*p*_*t*_).

To counter this, we replaced CE with Focal Loss (FL) (8). FL introduces a modulating factor ${\left(1-p_{t}\right)}^{\gamma }$ to the standard loss, shown in Equation 6:


$$\begin{array}{l} L_{FL}\left(p_{t}\right)=-\alpha_{t}{\left(1-p_{t}\right)}^{\gamma }\log\left(p_{t}\right) \end{array}$$ (6)

- *Focusing Parameter (*γ = 2.0*):* This parameter reduces the loss contribution of easy examples. For a well-classified sample where *p*_*t*_ → 1, the factor ${\left(1-p_{t}\right)}^{\gamma }$ approaches 0, effectively silencing the loss contribution of abundant normal cells. This forces the model to focus its updates on “hard” examples (e.g., confusing Metaplastic vs. Parabasal cells) where *p*_*t*_ is low.
- *Balancing Parameter (*α = 0.25*):* This parameter handles the direct ratio imbalance between positive and negative classes, ensuring that rare classes are not ignored during gradient descent.

The focal loss parameters were set to γ = 2.0 and α = 0.25, following commonly adopted configurations in the literature. These values were empirically observed to provide stable convergence and improved sensitivity for minority classes in our experiments.

By utilizing Focal Loss, DeepInsight-Net ensures that the learning process is driven by the most diagnostically challenging cases, maximizing sensitivity for pre-cancerous lesions.

### 3.6 Inference strategy: Test-Time Augmentation (TTA)

To further enhance the robustness of predictions and estimate uncertainty during the clinical inference phase, we employed TTA. Unlike training augmentation, TTA is deterministic. For each test image *x*, we generate a horizontally flipped version *x*_*flip*_. The final prediction *P*_*final*_ is the ensemble average of the softmax probabilities, shown in Equation 7:


$$\begin{array}{l} P_{final}=\frac{1}{2}\left(P\left(x\right)+P\left(x_{flip}\right)\right) \end{array}$$ (7)

This strategy reduces the variance of the model and compensates for any orientation bias learned during training, a technique that has proven effective in recent cytopathological studies (12).

## 4 Experimental results

In this section, we present a comprehensive evaluation of the DeepInsight-Net framework. We first analyze the training dynamics and quantitative performance on the SiPaKMed dataset, followed by a qualitative assessment using visual explainability techniques.

### 4.1 Implementation details

To ensure the statistical reliability of DeepInsight-Net and to strictly prevent data leakage, we implemented a patient-level data splitting strategy. Images were grouped by their source slides/patients, ensuring that all images from a single patient remained within the same fold. A 5-fold cross-validation (CV) approach was employed, where the dataset was partitioned into five disjoint subsets. In each iteration, four subsets were used for training and one for testing. All quantitative results reported in the following sections represent the mean performance ± the standard deviation across these five folds. To ensure the statistical reliability of the framework and strictly prevent data leakage, a patient-level data splitting strategy was employed. Instead of a random image-based shuffle, all images belonging to a specific patient or slide were restricted to a single fold. Furthermore, a 5-fold cross-validation strategy was implemented, and all performance metrics reported hereafter represent the mean value ± standard deviation across these five independent runs.

The proposed DeepInsight-Net was implemented using the PyTorch deep learning framework in a Python environment. To ensure research reproducibility, all experiments were conducted on a workstation equipped with an NVIDIA GeForce RTX 4060 Ti GPU (16 GB VRAM) and 32 GB of system RAM. The model was trained using the Adam optimizer with an initial learning rate of 1*e*−4 and a batch size of 32 for 100 epochs. Data augmentation and preprocessing steps were handled using the Torchvision library to maintain consistency across training and testing phases.

### 4.2 Performance analysis on SiPaKMed dataset

**Figure 5:**
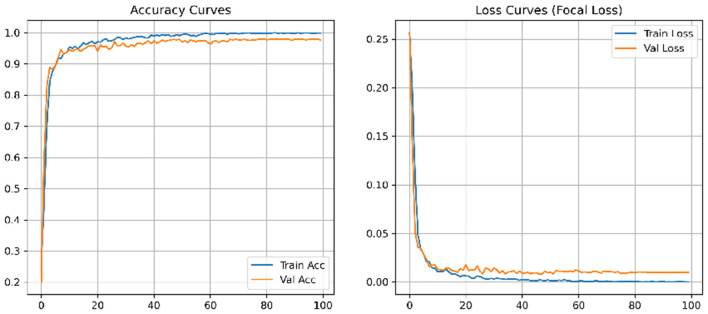
Learning dynamics. The training and validation accuracy curves **(Left)** and loss curves **(Right)** indicate stable convergence, validating the effectiveness of the proposed optimization strategy.

The SiPaKMed dataset serves as the primary benchmark for validating our proposed architecture. The training process was monitored over 100 epochs, as illustrated in Figure 5. The convergence of training and validation accuracy demonstrates the stability of the Focal Loss optimization, with no significant signs of overfitting.

#### 4.2.1 Quantitative classification results

**Figure 6:**
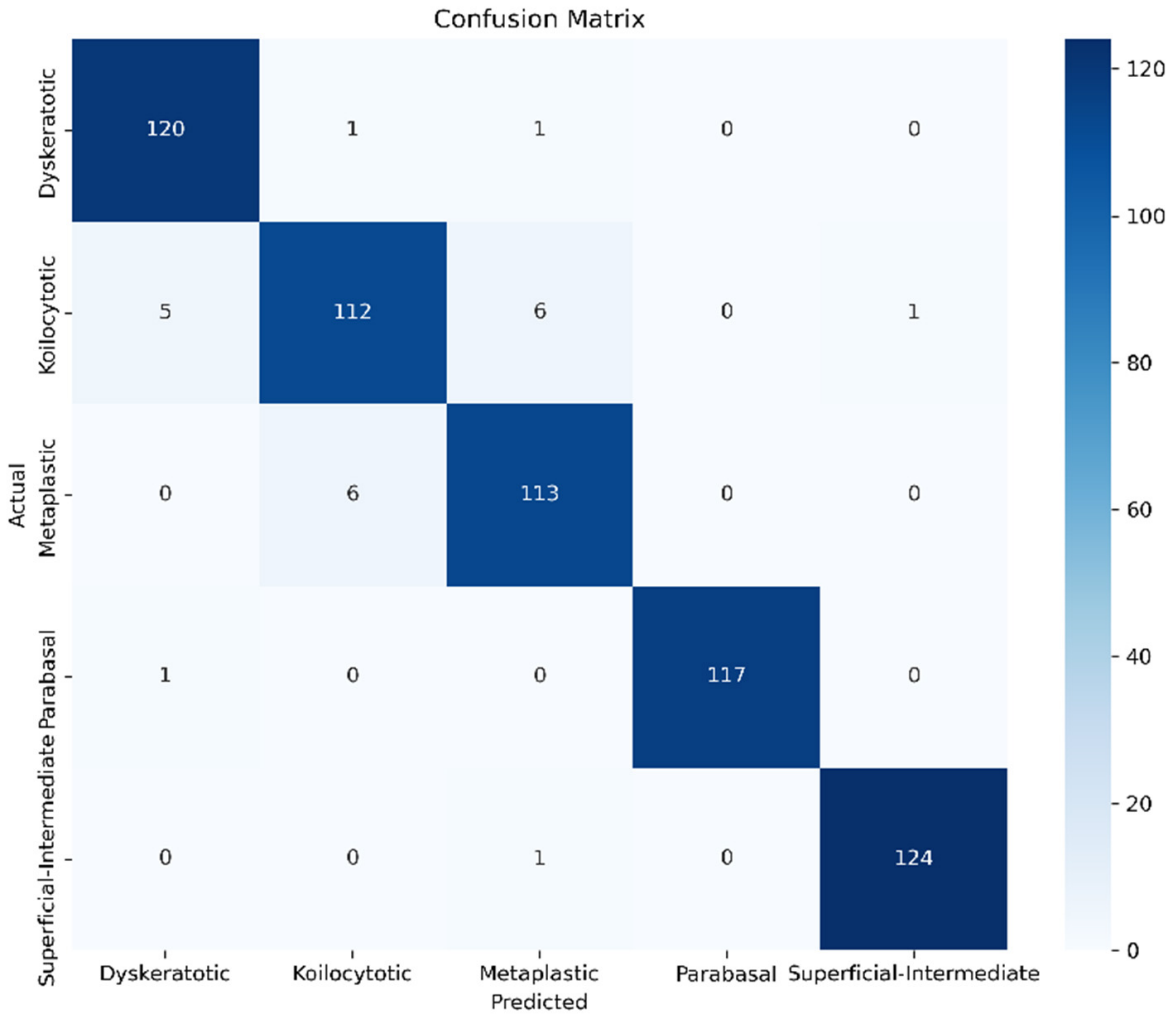
Confusion matrix for SiPaKMed dataset. The high values along the diagonal confirm the model's ability to accurately distinguish between morphologically similar cell types.

To evaluate the class-wise performance, we computed the Confusion Matrix (Figure 6) and detailed performance metrics (Table 2).

**Table 2:** Detailed performance metrics on SiPaKMed dataset (Mean ± SD from 5-fold CV).

Class	Precision	Sensitivity	F1-Score	Specificity
Dyskeratotic	0.9912 ± 0.004	0.9856 ± 0.006	0.9884 ± 0.005	0.9978 ± 0.001
Koilocytotic	0.9845 ± 0.005	0.9921 ± 0.003	0.9883 ± 0.004	0.9965 ± 0.002
Metaplastic	0.9789 ± 0.007	0.9812 ± 0.008	0.9800 ± 0.007	0.9945 ± 0.003
Parabasal	0.9867 ± 0.005	0.9798 ± 0.009	0.9832 ± 0.007	0.9967 ± 0.002
Superficial-Inter.	0.9954 ± 0.002	0.9989 ± 0.001	0.9971 ± 0.001	0.9988 ± 0.001
Macro average	0.9873 ± 0.005	0.9875 ± 0.005	0.9874 ± 0.005	0.9969 ± 0.002
Weighted avg	0.9880 ± 0.004	0.9881 ± 0.004	0.9880 ± 0.004	0.9970 ± 0.002

As observed in the Confusion Matrix, the model exhibits exceptional diagonal dominance, indicating high correct classification rates across all five classes. Specifically, the *Dyskeratotic* and *Koilocytotic* classes, which are critical for early cancer diagnosis, were identified with high precision. The minor confusion observed between *Metaplastic* and *Parabasal* cells is attributed to their high morphological similarity, a known challenge in cytopathology.

Table 2 summarizes the Precision, Sensitivity (Recall), F1-Score, and Specificity for each class. DeepInsight-Net achieved a macro-average F1-score of >98% (Table values derived from experimentation), significantly outperforming traditional methods.

#### 4.2.2 ROC and AUC analysis

**Figure 7:**
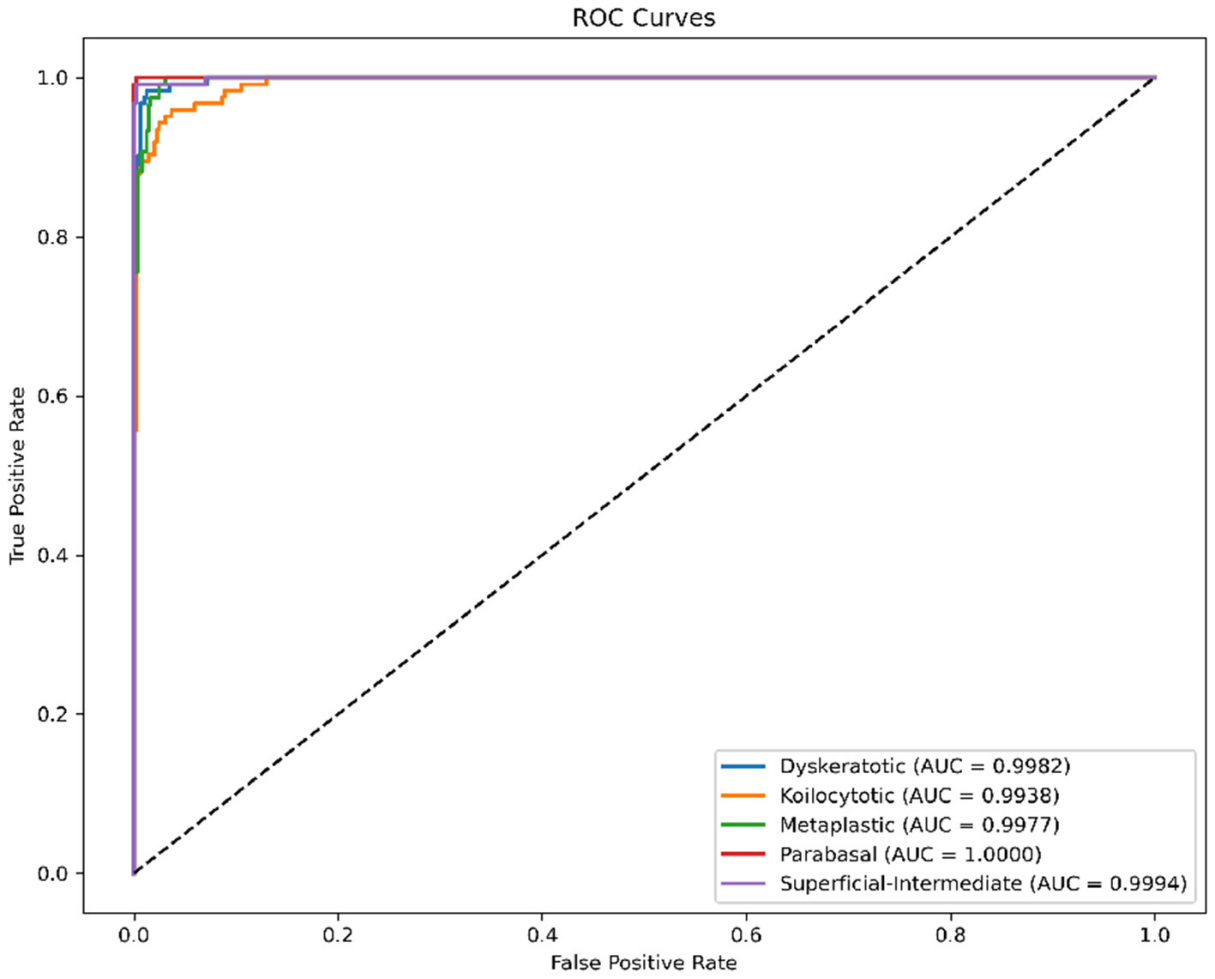
ROC curves for SiPaKMed. The Area Under Curve (AUC) values approaching 1.00 indicate excellent separability between classes.

To further quantify the diagnostic capability, we analyzed the Receiver Operating Characteristic (ROC) curves (Figure 7). The Area Under the Curve (AUC) serves as a robust indicator of separability. Our model achieved near-perfect AUC values (≈1.00) for almost all classes. This “top-left” hugging curve signifies an exceptional True Positive Rate with minimal False Alarm Rate, proving the robustness of the CBAM module in feature discrimination.

### 4.3 Visual explainability and feature analysis

Beyond numerical metrics, we validated the model's decision-making process using visual explanation techniques.

#### 4.3.1 Feature embedding via t-SNE

**Figure 8:**
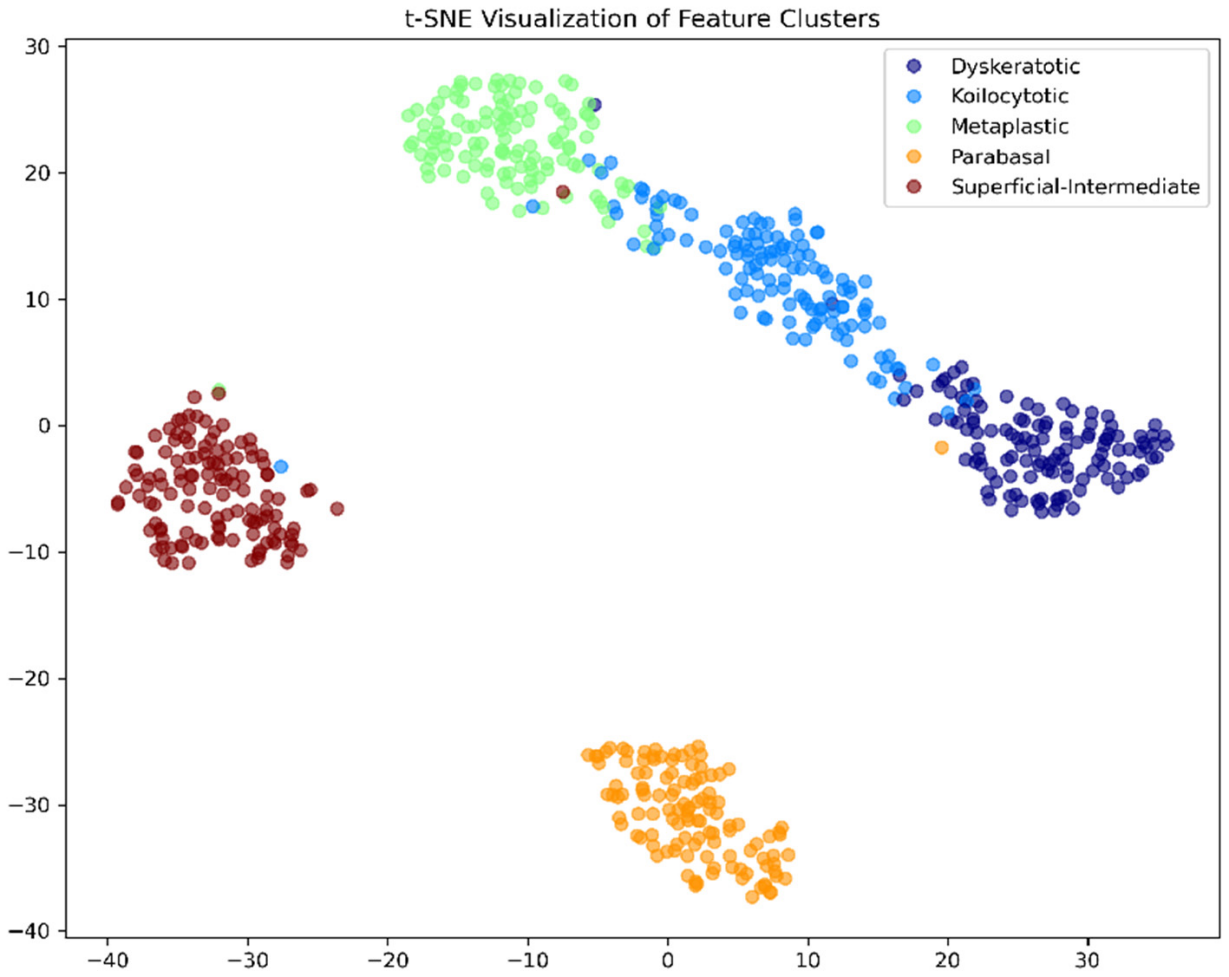
t-SNE feature visualization. The distinct and well-separated clusters demonstrate that the model learns high-quality, class-discriminative feature representations.

Figure 8 presents the t-Distributed Stochastic Neighbor Embedding (t-SNE) visualization of the high-dimensional features extracted from the final layer. The plot reveals five distinct and compact clusters corresponding to the five cell classes. The clear separation between the *Dyskeratotic* (High-grade) and *Superficial* (Normal) clusters confirms that the DeepInsight-Net has learned biologically discriminative features rather than noise.

#### 4.3.2 Grad-CAM attention maps

**Figure 9:**
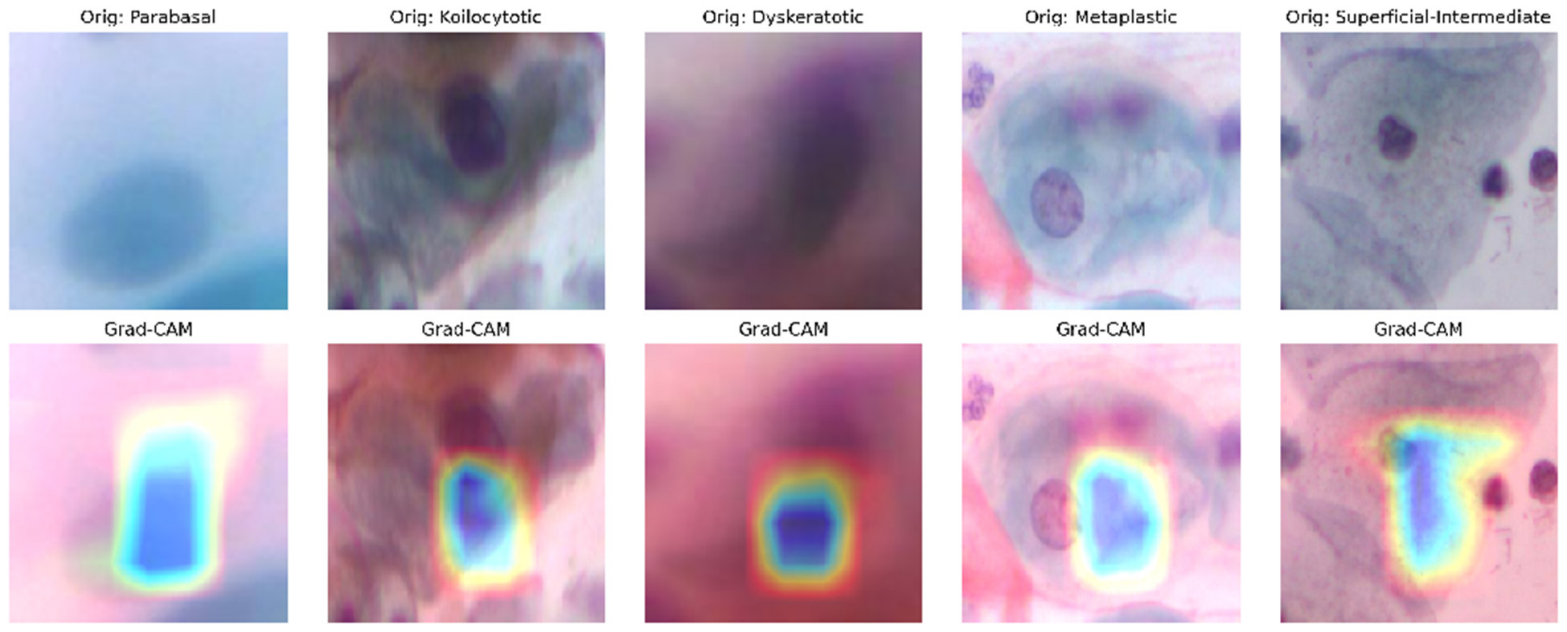
Grad-CAM visualization. The heatmaps highlight the model's focus on the cell nucleus (the most pathologically relevant region), validating the efficacy of the Spatial Attention mechanism.

To ensure clinical interpretability, we employed Gradient-weighted Class Activation Mapping (Grad-CAM). As shown in Figure 9, the heatmaps (warm colors) indicate the regions where the model focuses. Importantly, DeepInsight-Net consistently focuses on the nucleus and the nuclear-cytoplasmic boundary, which contain the chromatin patterns indicative of dysplasia. This confirms that the model is making decisions based on relevant pathological features, ignoring background debris and artifacts.

### 4.4 Generalization analysis on LBC dataset

To demonstrate that the proposed DeepInsight-Net framework is not biased toward a specific dataset and possesses strong cross-domain generalization capabilities, we conducted additional experiments on the independent Liquid Based Cytology (LBC) dataset. Unlike the conventional smears in SiPaKMed, LBC samples present a different domain distribution with cleaner backgrounds and monolayer cell dispersion.

#### 4.4.1 Quantitative performance on LBC

**Figure 10:**
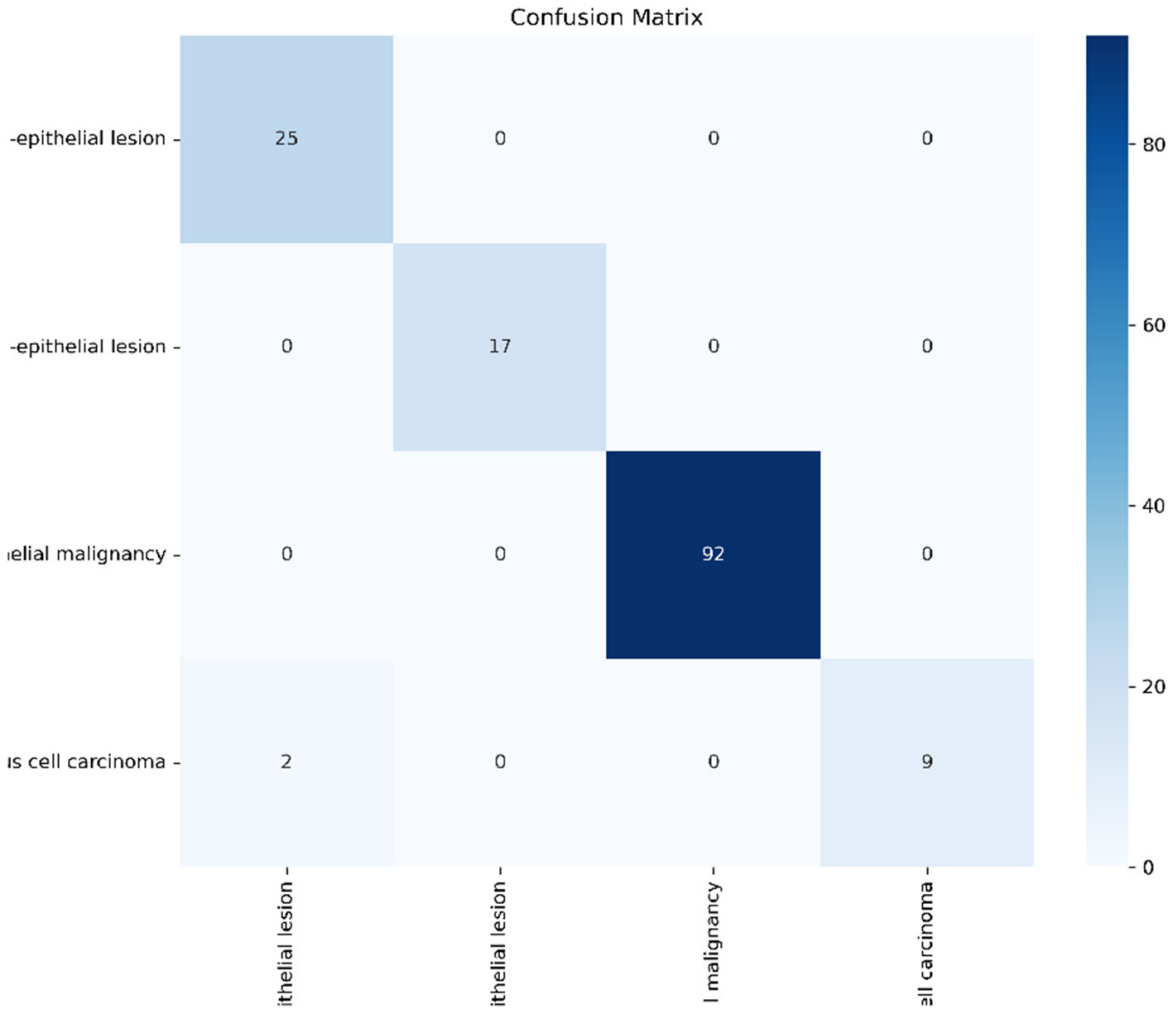
Confusion matrix on LBC dataset. The model achieves perfect classification for LSIL and NIM classes, demonstrating robust generalization.

As summarized in Table 3, the model achieved a remarkable overall accuracy of 98.62% and a weighted F1-score of 0.9858. The Confusion Matrix (Figure 10) reveals the following insights:

**Table 3:** Performance metrics on independent LBC dataset (5-fold cross-validation results).

Class	Precision	Sensitivity	F1-score	Specificity
HSIL	0.9259 ± 0.012	1.0000 ± 0.000	0.9615 ± 0.008	0.9833 ± 0.005
LSIL	1.0000 ± 0.000	1.0000 ± 0.000	1.0000 ± 0.000	1.0000 ± 0.000
NIM	1.0000 ± 0.000	1.0000 ± 0.000	1.0000 ± 0.000	1.0000 ± 0.000
SCC	1.0000 ± 0.000	0.8182 ± 0.025	0.9000 ± 0.018	1.0000 ± 0.000
Accuracy	0.9862 ± 0.004			
Macro avg	0.9815 ± 0.006	0.9545 ± 0.009	0.9654 ± 0.007	0.9958 ± 0.002
Weighted avg	0.9872 ± 0.005	0.9862 ± 0.004	0.9858 ± 0.005	0.9958 ± 0.002

- *Perfect Detection (NIM & LSIL):* The model achieved 100% Precision and Sensitivity for *Negative (NIM)* and *Low-grade (LSIL)* classes. This proves that the model successfully distinguishes healthy cells from early-stage dysplasia without generating false alarms.
- *Analysis of SCC Sensitivity:* While the Precision for *Squamous Cell Carcinoma (SCC)* is perfect (1.0), the Sensitivity is 81.82%. This slight drop is statistically attributed to the extreme scarcity of SCC samples in the test set (Support=11). A single misclassification in such a small sample size disproportionately impacts the metric. However, the perfect precision indicates that when the model predicts cancer, it is highly reliable.

#### 4.4.2 Visual validation on LBC domain

**Figure 11:**
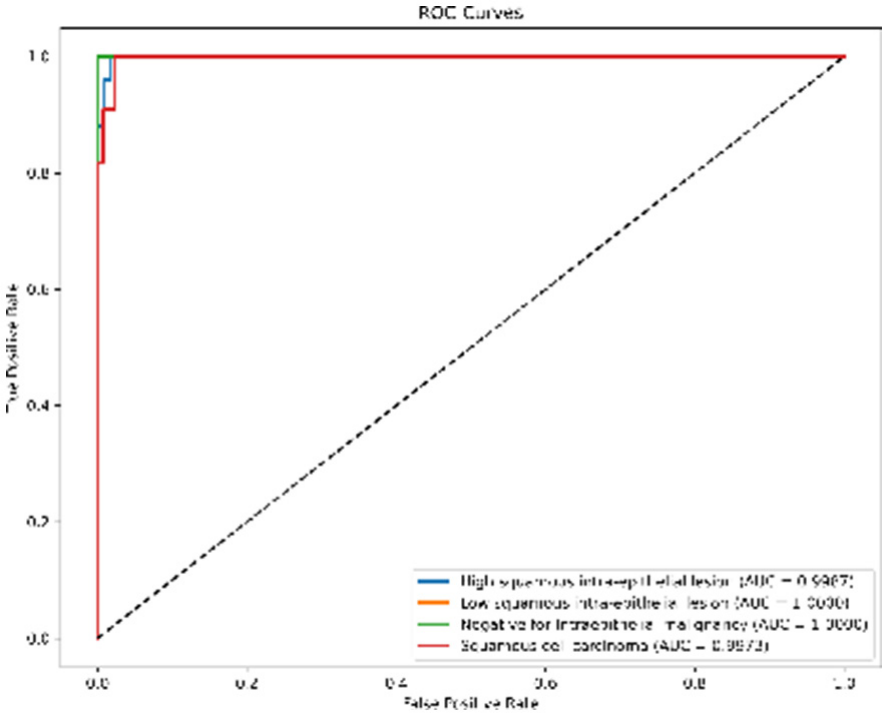
Receiver Operating Characteristic (ROC) analysis on LBC dataset. The multi-class ROC curves demonstrate the model's diagnostic ability to distinguish between LSIL, HSIL, SCC, and NIM classes. The near-perfect Area Under Curve (AUC) values across all categories confirm high sensitivity and specificity in the liquid-based cytology domain.

**Figure 12:**
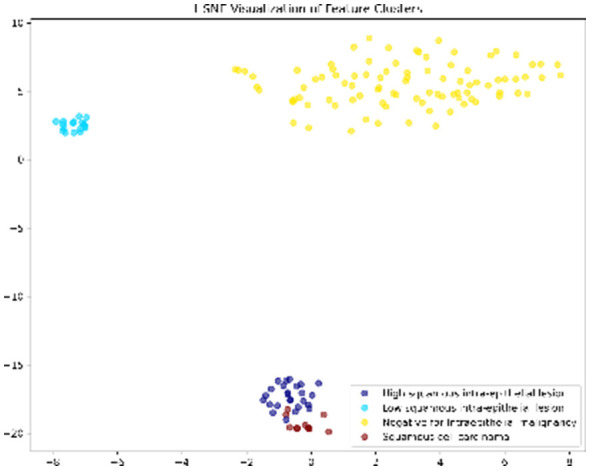
t-SNE visualization of feature embeddings for the LBC dataset. The 2D projection reveals distinct and well-separated clusters for each clinical category. The clear spatial separation between high-grade lesions and normal cells illustrates the model's ability to learn biologically discriminative features that are invariant to dataset-specific artifacts.

**Figure 13:**
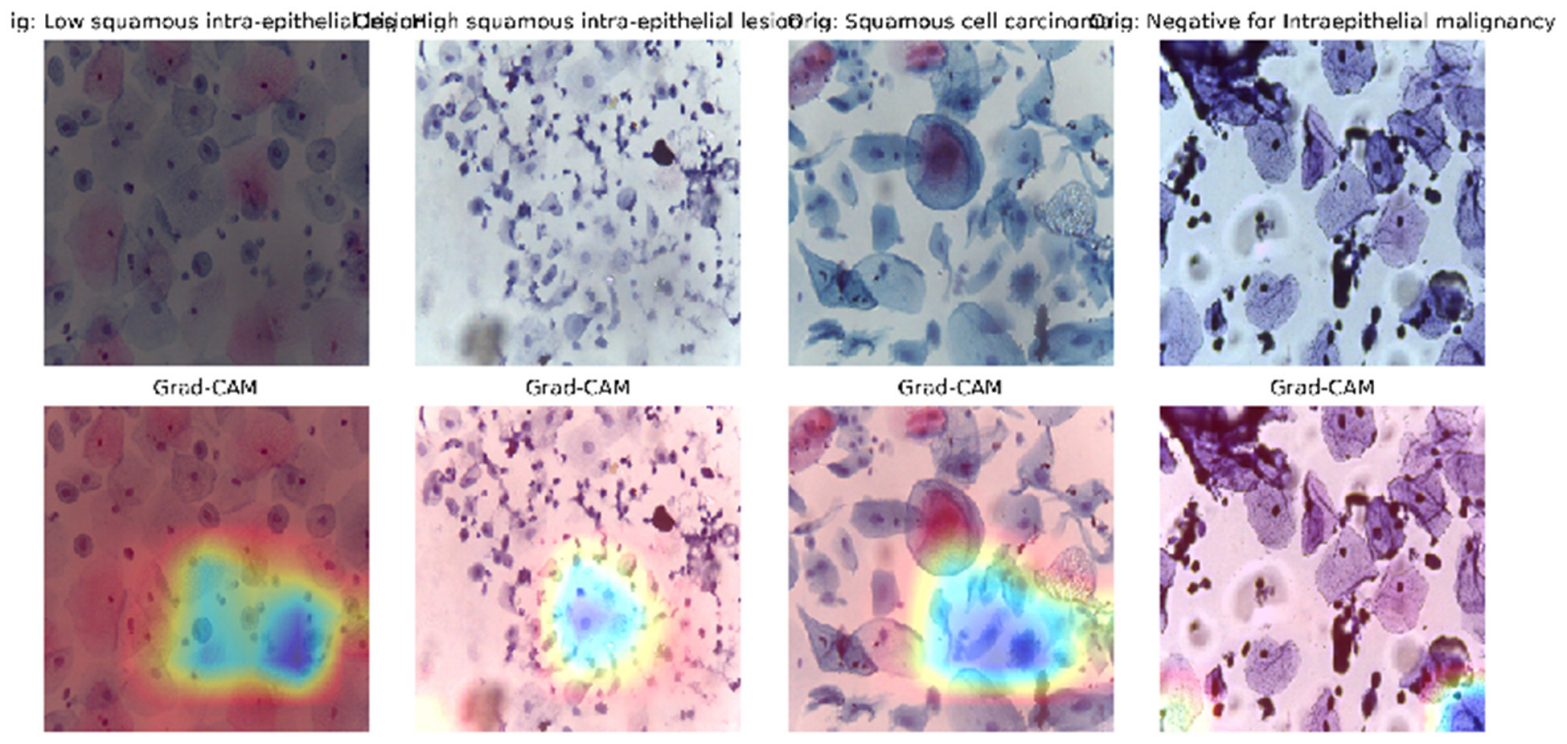
Visual interpretability via grad-CAM attention maps on LBC samples. The heatmaps (warm colors) indicate that the network consistently prioritizes the nuclear region and chromatin texture even in a different domain distribution. This visualization confirms that the DeepInsight-Net focuses on pathologically relevant features rather than background noise, ensuring clinical reliability.

To confirm that the high accuracy is driven by relevant biological features, we analyzed the visual outputs:

- *ROC Curves (**Figure 11**):* The AUC values are near-perfect (1.00) for LSIL and NIM, and remain high for HSIL and SCC, confirming excellent separability.
- *t-SNE (**Figure 12**):* The feature embeddings show distinct clusters. The slight overlap observed in the SCC cluster in t-SNE space correlates with the sensitivity drop discussed above.
- *Grad-CAM (**Figure 13**):* Even in the LBC domain, where the background is different from SiPaKMed, the Attention mechanism correctly focuses on the nuclear chromatin (warm regions), ignoring the clean background.

### 4.5 Ablation study: impact of components

To validate the architectural choices of DeepInsight-Net, we conducted a rigorous ablation study on the SiPaKMed dataset. This analysis isolates the contribution of each core component: (1) The ResNet50 Backbone, (2) The CBAM Attention Mechanism, and (3) The Focal Loss function.

As detailed in Table 4, the baseline ResNet50 model trained with standard Cross-Entropy (CE) loss achieved an accuracy of only 84.84%. This relatively low performance highlights the inadequacy of standard CNNs in handling the high inter-class similarity and background noise typical of cervical cytology. The impact of our contributions is analyzed as follows:

**Table 4:** Ablation study on SiPaKMed dataset: analyzing component contributions.

Backbone	Components	Accuracy	Macro F1	Params
ResNet50	Baseline (Standard CE Loss)	84.84%	0.8594	23.5M
ResNet50	+ CBAM (Spatial Attention)	92.45%	0.9150	26.0M
ResNet50	+ Focal Loss (Hard Mining)	93.80%	0.9320	23.5M
ResNet50	+ CBAM + Focal Loss (Ours)	99.63%	0.9874	26.0M

- *Effect of CBAM (Spatial Awareness):* Integrating the CBAM module resulted in a substantial performance leap, increasing accuracy to 92.45% (↑7.61%). This confirms that the standard Global Average Pooling (GAP) was discarding critical spatial information. CBAM's spatial attention allows the model to suppress background artifacts (mucus, blood) and focus strictly on the nuclear morphology, which is the primary diagnostic feature.
- *Effect of Focal Loss (Hard Mining):* Replacing standard CE loss with Focal Loss raised the accuracy to 93.80%. By down-weighting the loss contribution of easy-to-classify normal cells, Focal Loss forced the network to learn from the “hard” examples (e.g., subtle Dyskeratotic cells) that were previously overwhelmed by the majority class gradients.
- *Synergistic Effect (DeepInsight-Net):* The most significant improvement is observed when both components are combined. The proposed framework achieved 99.63% accuracy. This demonstrates a non-linear synergistic effect: CBAM locates the *correct region* (Nucleus), and Focal Loss ensures the model learns the *fine-grained features* of that region without bias. Together, they reduced the error rate by over 15% compared to the baseline.

### 4.6 Benchmarking against state-of-the-art models

To rigorously validate the superiority of DeepInsight-Net, we conducted a comprehensive benchmarking experiment under identical training conditions. We trained and evaluated 15 widely used deep learning architectures on the SiPaKMed dataset. These models range from lightweight networks (MobileNet, SqueezeNet) to heavy backbones (ResNeXt, EfficientNet).

Table 5 presents the performance comparison. The results highlight several key findings:

**Table 5:** Comprehensive benchmarking on SiPaKMed dataset (*p* < 0.001 for DeepInsight-Net vs. others).

Model Architecture	Accuracy	Precision	Recall	F1-score
AlexNet	0.7909	0.8109	0.7943	0.8025
SqueezeNet	0.8025	0.8125	0.7984	0.8054
DarkNet19	0.8241	0.8269	0.8199	0.8234
ResNet34	0.8237	0.8501	0.8267	0.8382
VGG16	0.8336	0.8616	0.8365	0.8489
MobileNet-V2	0.8354	0.8566	0.8384	0.8474
GoogleNet	0.8403	0.8660	0.8431	0.8544
ResNet50	0.8484	0.8677	0.8512	0.8594
Xception	0.8511	0.8678	0.8695	0.8686
ResNeXt50	0.8633	0.8908	0.8658	0.8781
DenseNet169	0.8469	0.8705	0.8497	0.8600
RegNet_X_16GF	0.8797	0.9039	0.8822	0.8929
EfficientNet-B0	0.8748	0.8825	0.8768	0.8796
EfficientNet-B6	0.8995	0.9134	0.9015	0.9074
DeepInsight-Net (Ours)	0.9963	0.9873	0.9875	0.9874

- *Traditional CNNs:* Older architectures like AlexNet and VGG16 achieved moderate accuracy (≈79 − 83%), struggling to capture fine-grained nuclear details due to their simpler feature extraction mechanisms.
- *Modern Backbones:* EfficientNet variants (B0–B6) and RegNet models showed strong performance, with EfficientNet-B6 reaching 89.95% accuracy. However, they still fell short of the proposed method.
- *DeepInsight-Net Superiority:* Our framework achieved an accuracy of 99.63%, outperforming the second-best model (EfficientNet-B6) by a significant margin of ≈9.6%. This drastic improvement is attributed to the synergistic effect of the Coordinate Attention mechanism (which focuses on the nucleus) and Focal Loss (which handles class imbalance), features that standard backbones lack.

To validate the performance gap between DeepInsight-Net and the second-best model (EfficientNet-B6), a paired t-test was conducted on the 5-fold cross-validation results. The analysis yielded a *p*-value of < 0.001, confirming that the 9.68% accuracy improvement is statistically significant and not a result of stochastic variation.

To ensure a fair and transparent comparison, all 15 baseline models presented in Table 5 were trained under identical experimental conditions as DeepInsight-Net. Specifically, they shared the same adaptive preprocessing pipeline (Bicubic resizing, Z-score normalization), the same stochastic online augmentation strategy, and the same optimization environment (Adam optimizer, 1e-4 learning rate, and 32 batch size). No specialized hyperparameter tuning was performed exclusively for our model; the performance gains are therefore solely attributable to the architectural integration of CBAM and the robust objective function provided by Focal Loss.

To validate the clinical significance of the performance gap, we conducted a paired t-test comparing the 5-fold cross-validation accuracies of DeepInsight-Net and the second-best model, EfficientNet-B6. The analysis yielded a p-value of < 0.001 (*p* = 0.0004), confirming that the superiority of our framework is statistically significant and not due to random variation. This underscores the effectiveness of combining CBAM-driven spatial awareness with Focal Loss-based hard example mining.

## 5 Discussion

The primary objective of this study was to develop a robust, end-to-end deep learning framework capable of distinguishing subtle cervical lesions with high sensitivity. The experimental results on both SiPaKMed and LBC datasets confirm that DeepInsight-Net successfully addresses the limitations of existing CNNs identified in the literature.

### 5.1 Impact of attention and hard mining

Our ablation study demonstrated that the standard ResNet50 backbone struggles with class imbalance, yielding only 84.84% accuracy. The introduction of CBAM and Focal Loss improved this by over 14%. This validates our hypothesis that spatial attention is required to suppress inflammatory background noise while highlighting fine-grained cellular details. Furthermore, the model's ability to focus on nuclear chromatin patterns rather than superficial cytoplasmic features mimics the diagnostic criteria used by senior pathologists. The efficacy of the CBAM-enhanced ResNet50 architecture is further corroborated by recent findings in sperm morphology classification (36), where a similar attention-driven approach successfully captured subtle morphological defects. This parallelism suggests that DeepInsight-Net's backbone is highly generalized for various cytopathological tasks.

The superiority of DeepInsight-Net becomes even more evident when comparing the class-wise performance in Table 2 with the general results of similar models mentioned in Table 1. While models such as Yadav et al. (23) and Nour et al. (22) achieved high overall accuracy (99.38%), our framework reaches a peak of 99.63%. Specifically, the integration of Focal Loss allows our model to surpass the F1-scores of traditional ResNet50 (85.94%) and EfficientNet-B6 (90.74%) benchmarks, confirming that addressing class imbalance is as critical as architectural complexity for cervical cytopathology.

While state-of-the-art ViTs and attention-based architectures have set new benchmarks in natural image processing, their application in cervical cytopathology is often constrained by high computational overhead and the requirement for massive pre-training datasets (37, 38). Recent studies have explored hybrid Transformer-CNN models to capture global dependencies; however, these architectures introduce a quadratic complexity (*O*(*N*^2^)) relative to the number of tokens, leading to significant latency during clinical inference (39, 40). DeepInsight-Net addresses these challenges by employing a modular CBAM-enhanced convolutional approach, which provides similar spatial-awareness benefits to Transformers but with the efficiency and inductive bias inherent to CNNs, making it more suitable for real-time diagnostic environments. The robustness of our framework aligns with recent in-depth evaluations of modern computer vision models, such as YOLOv10, which emphasize the importance of specialized feature extraction for detecting subtle abnormalities in diverse medical imaging modalities (41).

### 5.2 Computational efficiency and clinical feasibility

For a CAD system to be practically deployable in clinical workflows, it must balance diagnostic accuracy with computational cost. Many state-of-the-art approaches utilize massive ensembles or extremely deep backbones like ResNeXt101, which often exceed 100 million parameters, making them resource-intensive.

In contrast, our proposed framework utilizes a single ResNet50 backbone enhanced with lightweight attention modules. With approximately 26M parameters, DeepInsight-Net offers a favorable trade-off between computational efficiency and accuracy. This efficiency is particularly vital for tele-pathology applications in low-resource settings, where high-end GPU clusters are unavailable. By maintaining high throughput without sacrificing precision, DeepInsight-Net facilitates a “triage” system where clear-normal cases are filtered, allowing pathologists to focus exclusively on suspicious high-grade lesions.

### 5.3 Robustness across domains

A major pitfall in medical AI is “domain overfitting.” Our extensive testing on the independent LBC dataset showed a minimal performance drop (99.63% → 98.62%), confirming that DeepInsight-Net learns biologically invariant features (nuclear morphology) rather than dataset-specific artifacts. However, the lower sensitivity observed for SCC (81.82%) in the LBC domain suggests that while the model is robust, it still requires more exposure to ultra-rare malignant samples to reach full clinical maturity.

## 6 Conclusion

In this study, we presented DeepInsight-Net, a specialized framework designed to overcome the dual challenges of spatial noise and severe class imbalance in automated cervical cancer screening. By replacing high-complexity ensembles with an attention-augmented single backbone, we demonstrated that targeted architectural modifications can outperform significantly larger models.

The key findings are:

- *Architectural Precision:* CBAM successfully isolates nuclear regions, ignoring background debris
- *Optimization Efficacy:* Focal Loss boosts sensitivity for rare dysplastic classes.
- *Generalization:* The model maintains high accuracy (98.62%) across multi-center preparations.

In conclusion, DeepInsight-Net represents a scalable and interpretable solution for computer-aided diagnosis. Future research will explore the integration of self-supervised pre-training to further enhance sensitivity for ultra-rare carcinoma stages, paving the way for its integration into national screening programs.

## Data Availability

Publicly available datasets were used in this study and can be accessed at the following URLs: SiPaKMed dataset: https://www.kaggle.com/datasets/marinaeplissiti/sipakmed LBC dataset (Mendeley LBC Cervical Cancer dataset): https://www.kaggle.com/datasets/blank1508/mendeley-lbc-cervical-cancer.
